# Optical absorption and photoluminescence studies of gold nanoparticles deposited on porous silicon

**DOI:** 10.1186/1556-276X-8-35

**Published:** 2013-01-18

**Authors:** Tengku Sarah Tengku Amran, Md Roslan Hashim, Nihad K Ali Al-Obaidi, Hanani Yazid, Rohana Adnan

**Affiliations:** 1 School of Physics, Universiti Sains Malaysia, Penang, 11800, Malaysia; 2 Material Innovations and Nanoelectronics (MINE) Research Group, Faculty of Electrical Engineering, Universiti Teknologi Malaysia, Skudai, Johor, 81310, Malaysia; 3 Ibnu Sina Institute for Fundamental Science Studies, Universiti Teknologi Malaysia, Skudai, Johor, 81310, Malaysia; 4 School of Chemical Sciences, Universiti Sains Malaysia, Penang, 11800, Malaysia

**Keywords:** Gold nanoparticles, Porous silicon, Deposition, Photoluminescence.

## Abstract

We present an investigation on a coupled system consists of gold nanoparticles and silicon nanocrystals. Gold nanoparticles (AuNPs) embedded into porous silicon (PSi) were prepared using the electrochemical deposition method. Scanning electron microscope images and energy-dispersive X-ray results indicated that the growth of AuNPs on PSi varies with current density. X-ray diffraction analysis showed the presence of cubic gold phases with crystallite sizes around 40 to 58 nm. Size dependence on the plasmon absorption was studied from nanoparticles with various sizes. Comparison with the reference sample, PSi without AuNP deposition, showed a significant blueshift with decreasing AuNP size which was explained in terms of optical coupling between PSi and AuNPs within the pores featuring localized plasmon resonances.

## Background

Coupling system involving semiconductor nanocrystals (NCs) and metal nanoparticles (NPs) has been a subject of great interest for the scientific community [1]. Due to the plasmon resonance in metal NPs, the interplay between NCs and NPs can modify the spectral features of NCs to improve emission efficiency as it involves the charge transfer across the semiconductor/metal interfaces [2]. Gold nanoparticles (AuNPs) are the subject of increasing interests due to their essential properties and localized surface plasmon resonance in the visible spectrum wavelength [3]. The interplay effect in combining the gold and silicon is widely used in electronic devices in controlling their lifetime and resistivity [4,5].

The AuNPs are mostly fabricated using a combination of chemical e-beam lithography and self-assembly techniques [6,7] or by electron beam evaporation [8]. However, the challenge is to control the size and position of the nanoparticles because these techniques tend to show a slightly broader size distribution. Mafuné et al. [9] have developed the laser ablation and laser-induced method to control the size of AuNPs without contamination. Nevertheless, this technique is very costly to implement. As an alternative, electrodeposition technique can offer a solution to the problems as it is known for its simplicity and low processing cost [10].

Instead of using silicon as the substrate for the AuNP deposition, Fukami et al. [11] discovered the use of porous Si to control the shape and alignment of metal nanostructures. In this paper, we demonstrate that AuNPs supported on zinc oxide (ZnO) that was synthesized via the deposition-precipitation method can be deposited into porous silicon (PSi) using electrochemical deposition (ECD) technique. The deposition-precipitation method has been proven to produce gold particles of size less than 5 nm [12]. The growth parameters such as pore size distribution of PSi, metal solution concentration, and exposure time may have major influence on the AuNP growth.

## Methods

### Preparation of porous silicon using pulsed technique

An n-type <100 > −oriented silicon wafer with a resistivity of 1 to 10 Ω cm was used to fabricate the PSi substrate. The substrate was cleaned in a wet chemical etching process, using RCA cleaning method. After cleaning, the samples were prepared using pulsed anodic etching method [13]. Output signal from the pulse current generator was used to feed the current at a constant peak of 10 mA/cm^2^ by adjusting the pause time (*T*_off_) at 4 ms with cycle time *T*_all_ (14 ms). The electrolyte solution used was a mixture of hydrofluoric acid and ethanol, 1:4 by volume. Anodization process was carried out for 30 min for all samples under illumination of a 100-W incandescent white light, 15 cm away from the samples. After the etching, the samples were rinsed in deionized water and dried in ambient air.

### Preparation of gold nanoparticle supported on zinc oxide

The AuNPs were prepared using the procedure basically similar to that described in our previous work [12] using deposition-precipitation method. The solution of 100 mL of HAuCl_4_ solution was heated to 80°C where the pH was adjusted by dropwise mixing with 0.5 M NaOH. Relatively, 1.00 g of zinc oxide support was immersed into the solution. In order to maintain the pH after the support was inserted, dropwise addition of 1.5 M HCl was prepared. The suspension was thermostated at 80°C and underwent vigorous stirring for 2 h. After that, the precipitates were washed with distilled water to remove residual sodium, chloride ions, and unreacted Au species. This process was repeated until there were no AgCl precipitates detected when a filter was added to the AgNO_3._ The resulting precipitate was gathered by centrifugation and dried at 100°C overnight. The calcination procedure was brought out at 450°C under ambient air for 4 h and a temperature gradient of 50°C min^−1^. About 0.6 g of black powder was finally obtained. The mean diameter of AuNPs less than 5 nm at pH 7 was obtained.

### Fabrication of AuNPs using electrochemical deposition method

The as-prepared Au nanoparticle powder was dispersed in aqua regia [14] and diluted with deionized water, forming yellow solutions with a mass concentration of approximately 2.8 mg/mL. The aqua regia was prepared by mixing one part of concentrated HNO_3_ with four parts of concentrated HCL to dissolve the gold. It was stirred using the hot plate magnetic stirrer at 20°C for 15 min. The solvent then was used in electrochemical deposition process using direct current at different current densities of 1.5, 2.5, 3.5, and 4.5 mA/cm^2^ for 30 min. Gold wire (99.999% purity) was an anode, and PSi was a cathode. The distance between the two electrodes was approximately about 0.5 cm. After that, the samples were dried under nitrogen flow and followed by annealing at 350°C for 15 min. The deposited samples were characterized using scanning electron microscopy (SEM), energy-dispersive X-ray spectroscopy (EDX), X-ray diffraction (XRD), and photoluminescence spectroscopy (PL).

## Results and discussion

### Transmission electron microscopy

**Figure 1:**
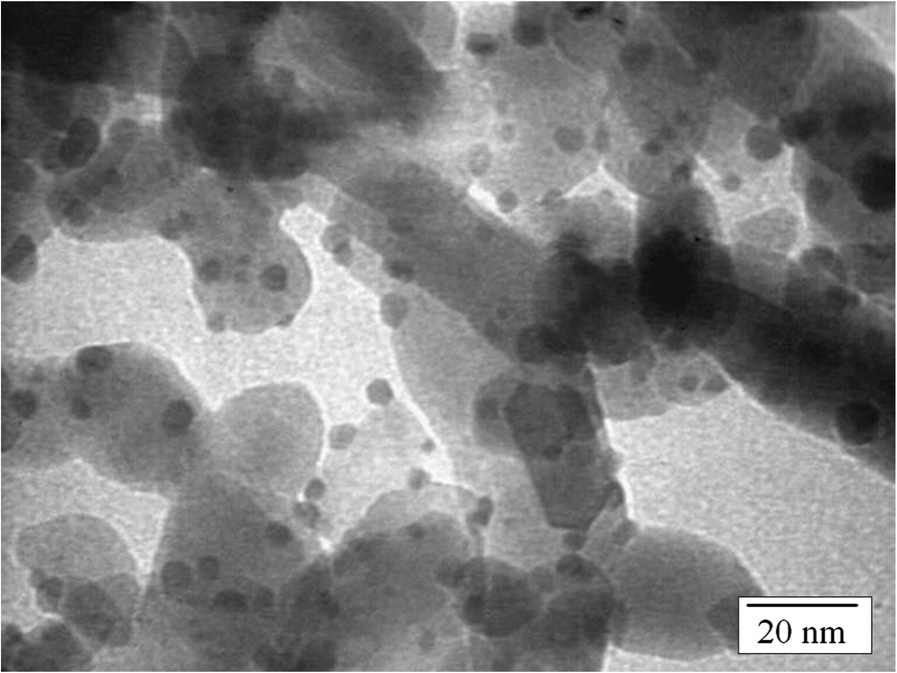
**TEM micrographs of Au/ZnO. **The average size less than 5 nm.

The gold images in the transmission electron microscopy (TEM) analysis (Figure 1) are represented by the small dark particles while the ZnO is shown as the larger particles with less intense color. The TEM images clearly show that the Au particles are deposited on the support. The average size of the Au particles is 4.45 ± 1.80 nm with 1- to 15-nm particle size distribution. It shows that the Au nanoparticles supported on ZnO prepared via the deposition-precipitation method produced average gold particle size less than 5 nm with maximal gold loading.

### Scanning electron microscopy

**Figure 2:**
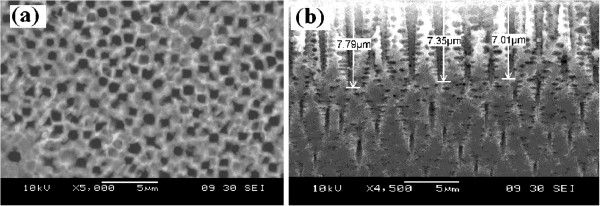
**SEM images of PSi. (a)** Top view of PSi etching using pulsed current method at a constant peak current density of 10 mA/cm^2^ with cycle time, *T*_all_, 14 ms and pulse time, *T*_off_, 4 ms and **(b)** cross section of the pores with estimated length of 7.4 ± 3 μm.

Figure 2a shows the top-view SEM image of the PSi formed using pulsed current method at a constant peak current density of 10 mA/cm^2^ with cycle time, *T*_all_ 14 ms and pulse time, *T*_off_ 4 ms. A uniform pore distribution is observed with estimated sizes around 2 ± 1 μm. Average pore depth of about 7.4 ± 3 μm and distinguished sharp pin-shaped holes are observed, as shown in Figure 2b.

**Figure 3:**
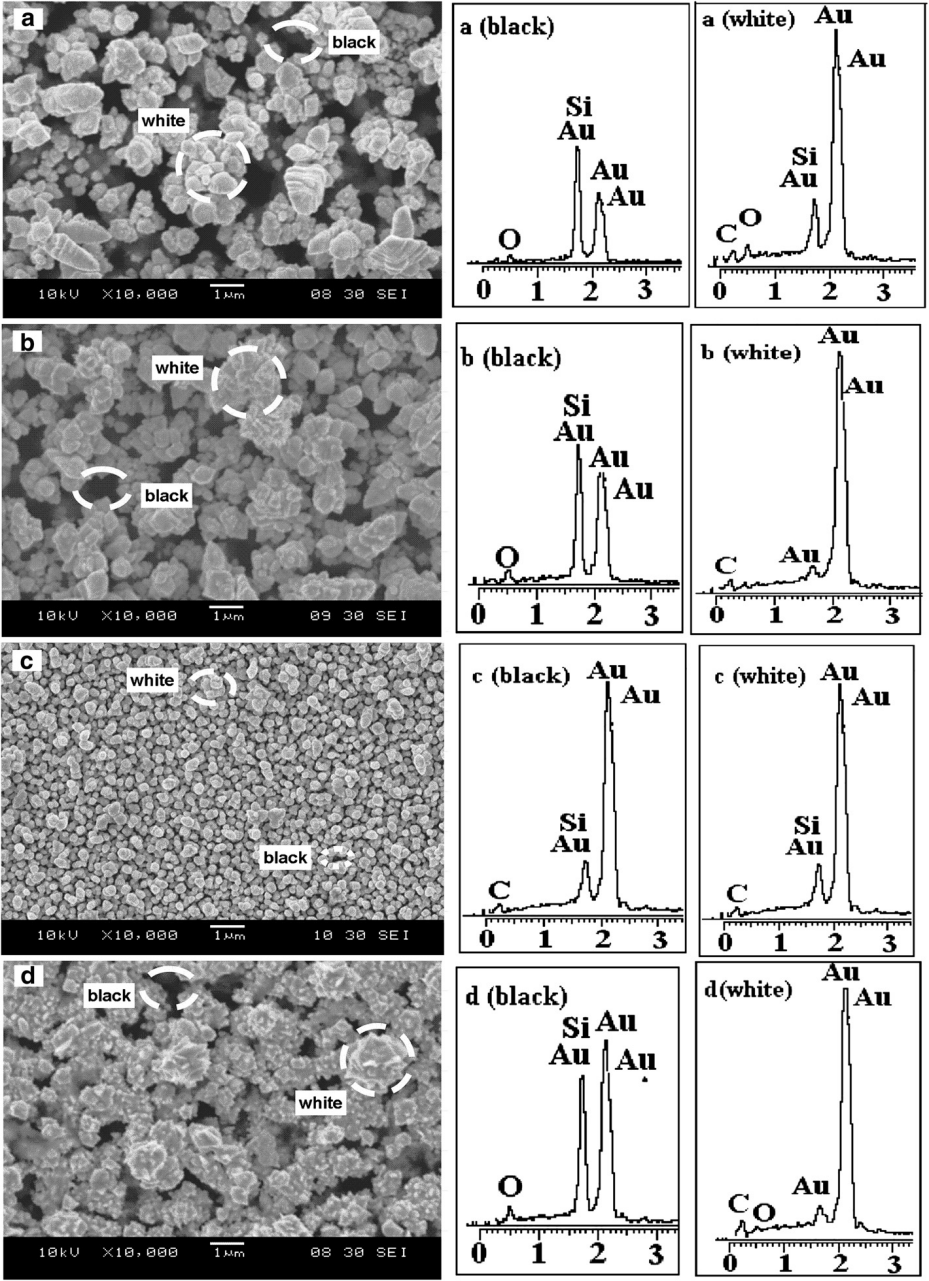
**SEM images with EDX spectra of Au/PSi. **The black and white spots in the SEM images and EDX spectrum for the sample PSi deposited with AuNPs at different current densities: **(a)** 1.5, **(b)** 2.5, **(c)** 3.5, and **(d)** 4.5 mA/cm^2^.

Figure 3 shows the SEM images and EDX spectrum of AuNPs deposited on PSi (Au/PSi) at different current densities of 1.5, 2.5, 3.5, and 4.5 mA/cm^2^ for 30 min. The images showed well-developed, faceted, large Au colloidal crystals prepared through the ECD method. The density of faceted grain sizes of AuNPs changes with current density. The Au colloidal crystal showed a mixture of large and small sizes from 100 nm to 2.0 μm for 1.5 mA/cm^2^ (Figure 3a), denser and wide distribution of larger grain sizes of Au particles with uniform sizes of 500 nm for 2.5mA/cm^2^ (Figure 3b), and smaller sizes, denser and more uniform AuNPs having estimated sizes ranging from 100 to 300 nm was observed for 3.5mA/cm^2^ (Figure 3c). The grain sizes became larger and more widely distributed around the surfaces for 4.5 mA/cm^2^ (Figure 3d), having homogeneous size distribution around 1.0 μm. The elemental composition of these faceted crystals is qualitatively determined using EDX spectroscopy. The EDX analysis was conducted on the white and black spots, which represent the gold and pores, respectively. The results showed that the significant Au peak appears from the black spot which is the pore area. This suggested that the AuNPs had diffused inside the pore of silicon nanostructures.

The potential reaction observed in dissolving the gold nanoparticle using aqua regia as an electrolyte for the ECD process can be expressed as follows [15]:

(1) $$\begin{array}{l} \;\mathrm{Au}\;\left(\text{s}\right)+{3\mathrm{NO}}_{3}{}^{-}\left(\mathrm{aq}\right)+{6\text{H}}^{+}\left(\mathrm{aq}\right)\to {\mathrm{Au}}^{3+}\left(\mathrm{aq}\right)\\ \;+{3\mathrm{NO}}_{2}\left(\text{g}\right)+{3\text{H}}_{2}O\; \end{array}$$

(2) $${\mathrm{Au}}^{3+}\left(\mathrm{aq}\right)+{4\mathrm{Cl}}^{-}\left(\mathrm{aq}\right)\to {\mathrm{AuCl}}_{4}{}^{-}\left(\mathrm{aq}\right)\text{.}$$

This resulted in a removal of positive gold ions (Au^3+^) from the solution and allowed further oxidation of gold to take place, and so, the gold was dissolved. In addition, Cl^−^ (from hydrochloric acid) removed Au^3+^ from the solution, encouraging NO^3−^ to dissolve a bit more gold. The longer the process goes on, the larger the Au particles become in size. We believe that there is an appropriate current density in which the formation of a high percentage of Au particle could be accelerated. Accordingly, from the SEM and EDX analyses, when the current density increases from 1.5 to 2.5 mA/cm^2^, the deposition rate of AuNPs increases. Consequently, at 3.5 mA/cm^2^, the deposition rate produces Au colloidal crystal with smaller sizes that widely distributed on the substrate. Beyond 3.5 mA/cm^2^, the deposition rate increases, and it enhances the fabrication of Au grain size. Thus, larger sizes of AuNPs were produced.

### X-ray diffraction

**Figure 4:**
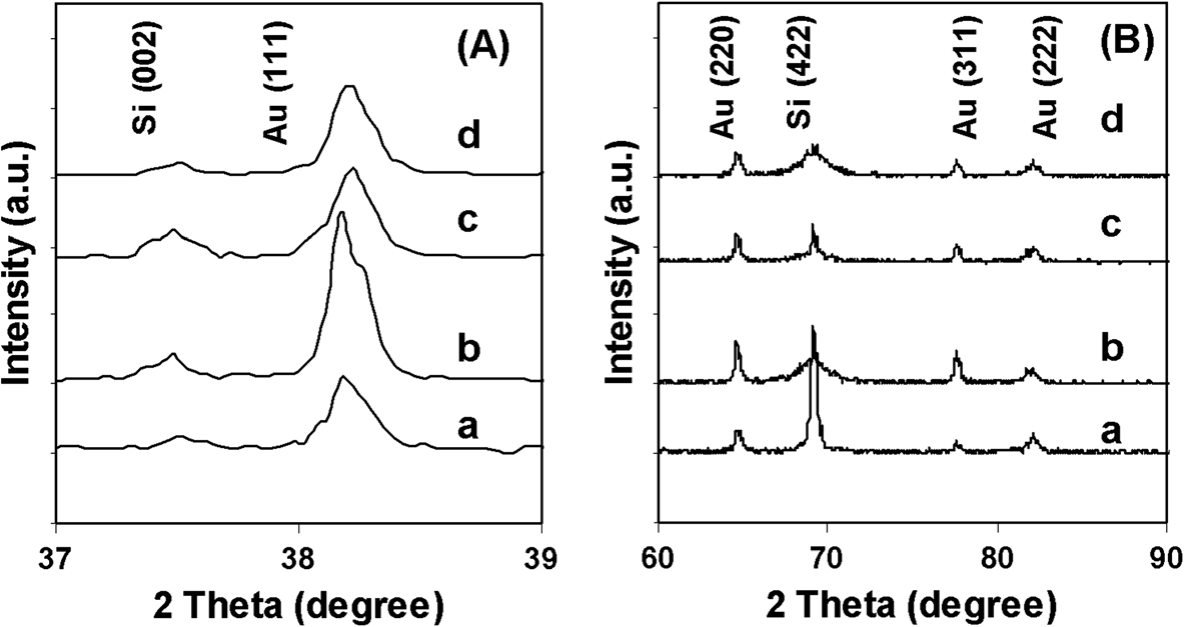
**XRD patterns of deposited Au/PSi. **Samples were deposited using different current densities of **(a)** 1.5, **(b)** 2.5, **(c)** 3.5, and **(d)** 4.5 mA/cm^2^, respectively, **(A)** for the range 2*θ* = 37° to 39° and **(B)** for the range of 2*θ =* 60° to 90°.

Figure 4 shows the typical XRD patterns of Au/PSi at different current densities. The XRD spectrum (Figure 4A) spectrum revealed two peaks: 2*θ* = 37.7° and 2*θ* = 38.2° for Si (002) and Au (111). A strong peak at 2*θ* = 38.2° indicates the higher population of Au (111) [16], which is the preferred orientation for the gold particle. Among the index facets of Au, Au (111) facet has the lowest surface energy. Thus, during chemical deposition, AuCl_4_^−^ ions will be preferentially absorbed on other index facets, and these absorbed AuCl_4_^−^ will be reduced to Au particle by the hydrochloric acid present in the solution. Therefore, the longer the processes go on, the whole substrates become enriched with Au (111) facet and become large in sizes. The XRD patterns of Au embedded into PSi (Figure 4B) revealed the diffraction peaks for cubic gold at 2*θ* = 64.6°, 77.5°, and 81.7°, which correspond to the crystal planes of (220), (311), and (222), respectively. The strong peak at 2*θ* = 69.5° is due to Si (422). The estimated Au crystallite size calculated using the Scherrer equation [17] from this peak is 58 nm for 1.5 mA/cm^2^, 50 nm for 2.5 mA/cm^2^, 51 nm for 3.5 mA/cm^2^, and 40 nm for 4.5 mA/cm^2^, respectively, indicating smaller crystallite size with increasing deposition current. The current density, angle (2*θ*), full width at half maximum (FHWM), and Au crystallite size are summarized in Table 1.

**Table 1:** The current density, angle (2*θ*), FHWM and Au crystallite size for all the samples

**Sample**	**Current density (mA/cm**^**2**^**)**	**Angle (2*θ*)**	**FHWM (2*θ*)**	**Crystallite size (nm)**
a	1.5	38.188	0.146	58
b	2.5	38.166	0.208	50
c	3.5	38.208	0.166	51
d	4.5	38.188	0.208	40

### Photoluminescence

**Figure 5:**
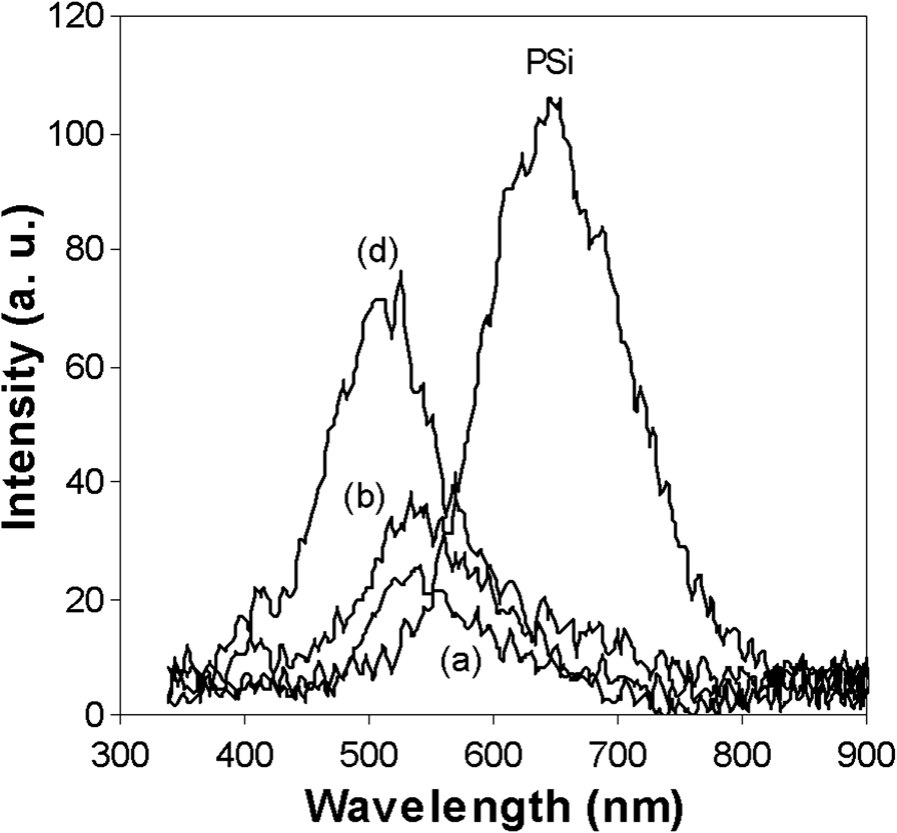
**The PL spectra of Au/PSi. **PL spectra for deposited AuNPs on PSi for 30 min using different current densities, **(a)** 1.5, **(b)** 2.5, and **(d)** 4.5 mA/cm^2^, and PSi as the reference.

Figure 5 shows the PL spectrum of PSi deposited with AuNPs at different current densities. The PL spectrum was characterized by the presence of one sharp peak in the red-band region showing the fundamental absorption of PSi (*E*_g_ = 1.91 eV) with the peak centered at 647 nm. It is attributed to the quantum confinement of electrons from Si nanocrystallites [18]. Another emission is observed with energy above the PSi bandgap around 2.34 eV (530 nm) showing broad and intense peak. PL spectrum in this region have different peak positions due to the formation of AuNPs of different sizes. We suggested that the origin of this band comes from the exciting laser that penetrated through the porous layer and directly exciting throughout AuNPs. Optical coupling between them is claimed to govern the observed behavior [19,20]. Meanwhile, the sample deposited with 3.5 mA/cm^2^ (sample c) had almost no PL peak that it is not included in the figure. The disappearance of the peak is consistent with the SEM image (Figure 3c) showing that the deposition of AuNPs with smaller grain sizes covered the entire PSi surface, which prevents the optical coupling process from taking place.

AuNPs exhibit the local electric field caused by the localized surface plasmon resonance behavior. When incident laser hits onto the surface, it caused the surface plasmon excitation and locally enhanced the electromagnetic field near the AuNPs and gave rise to PL emission. It has been known that the intensity of the of the plasmon peak is greatly dependent on the size and shape of AuNPs [20]. Therefore, as arranging them in crystallite size, we can see a general trend of increased emitted energy with decreasing crystallite size. Generally, as the gold crystallite size becomes smaller, the PL intensity becomes higher and stronger. Sample d shows the highest PL intensity by a factor of 4 compared to the other samples. It also noted that the plasmon peak exhibits blueshift with decreasing particle size. The observed blueshift in the peak position of plasmon absorption can be attributed to the quantum size effects from the AuNPs [21]. The optical properties of this composite structure, PSi containing AuNPs, reveal dielectric changes when the electromagnetic fields from AuNPs couple with PSi emission that leads to a clear blueshift (relative to the PSi sample) [22]. It is therefore noted that a current density of 4.5 mA/cm^2^ for electrodeposition produced the highest PL intensity.

## Conclusions

In conclusion, we have succeeded growing AuNPs on the surface of PSi using a cost-effective and simple method of electrochemical deposition. We showed that current density of the deposition process defined the size of the formed AuNPs. The surface morphology showed a gold colloidal crystal network with varying sizes. XRD spectra confirmed the element inside the Au/PSi whereby the peaks of Au (111) emerged as the preferred orientation. The samples contain cubic gold phase with crystallite size in the range of 40 to 58 nm. The blueshifted spectrum was caused by the interface interactions between AuNPs and the PSi, which are interpreted in terms of localized surface plasmon resonance of the AuNPs due to the change of the size condition which leads to a spectral shift of the spectrum in the PL.

## Competing interests

The authors declare that they have no competing interests.

## Authors’ contributions

TSTA carried out the main experimental work. MRH supervised the research activity. NKAA organized the manuscript. HY and RA prepared and made the chemical characterization of the AuNPs. All authors read and approved the final manuscript.

## Authors’ information

TSTA is a MSc student of the University Sains Malaysia (USM) together with HY. MRH is professor at the USM. NKA is the senior lecturer at the University Teknologi Malaysia (UTM). RA is an associate professor at USM.
